# In Vitro and In Vivo Multispectral Photoacoustic Imaging for the Evaluation of Chromophore Concentration

**DOI:** 10.3390/s21103366

**Published:** 2021-05-12

**Authors:** Aneline Dolet, Rita Ammanouil, Virginie Petrilli, Cédric Richard, Piero Tortoli, Didier Vray, François Varray

**Affiliations:** 1 Univ. Lyon, INSA-Lyon, Université Claude Bernard Lyon 1, UJM-Saint Etienne, CNRS, Inserm, CREATIS UMR 5220, U1206, F-69621 Lyon, France; aneline.dolet@grenoble-inp.fr (A.D.); didier.vray@creatis.insa-lyon.fr (D.V.); 2 Department of Information Engineering, University of Florence, 50139 Florence, Italy; piero.tortoli@unifi.it; 3 College of Engineering and Technology, American University of the Middle East, Kuwait, Kuwait; ritaammanouil@gmail.com; 4 CNRS UMR5286 INSERM U1052 Center de Recherche en Cancérologie de Lyon, F-69000 Lyon, France; virginie.petrilli@lyon.unicancer.fr; 5 Université de Lyon, Université Lyon 1, F-69000 Lyon, France; 6 Centre Léon Bérard, F-69008 Lyon, France; 7 Observatoire de la Côte d’Azur, CNRS, Laboratoire Lagrange, Université Côte d’Azur, 06300 Nice, France; cedric.richard@unice.fr

**Keywords:** photoacoustic imaging, spectral unmixing, blood oxygen concentration evaluation

## Abstract

Multispectral photoacoustic imaging is a powerful noninvasive medical imaging technique that provides access to functional information. In this study, a set of methods is proposed and validated, with experimental multispectral photoacoustic images used to estimate the concentration of chromophores. The unmixing techniques used in this paper consist of two steps: (1) automatic extraction of the reference spectrum of each pure chromophore; and (2) abundance calculation of each pure chromophore from the estimated reference spectra. The compared strategies bring positivity and sum-to-one constraints, from the hyperspectral remote sensing field to multispectral photoacoustic, to evaluate chromophore concentration. Particularly, the study extracts the endmembers and compares the algorithms from the hyperspectral remote sensing domain and a dedicated algorithm for segmentation of multispectral photoacoustic data to this end. First, these strategies are tested with dilution and mixing of chromophores on colored 4% agar phantom data. Then, some preliminary in vivo experiments are performed. These consist of estimations of the oxygen saturation rate (sO${}_{2}$) in mouse tumors. This article proposes then a proof-of-concept of the interest to bring hyperspectral remote sensing algorithms to multispectral photoacoustic imaging for the estimation of chromophore concentration.

## 1. Introduction

Photoacoustic imaging is a hybrid medical imaging technique that provides access to functional information. Nanosecond laser pulse illumination can provide optical properties of biological tissues that are related to molecular composition. The transmitted optical energy is absorbed by the optical absorbers in the tissues, which then undergo thermal expansion. At each laser pulse, this phenomenon generates ultrasonic waves that can be detected at the tissue surface by a conventional ultrasound system. The use of several laser wavelengths provides the multispectral absorption characteristics that can be used to distinguish the imaged tissues from each other [1,2]. In addition, as the absorption by a chromophore is linearly related to its concentration, multispectral photoacoustic imaging can also be used to determine the concentrations of chromophores present in the imaged region [3,4].

These two properties, discrimination of tissues and concentration evaluation, are of major interest, depending on the application at hand. They can be exploited to determine different concentrations of the same chromophore (e.g., estimation of the concentration of a contrast agent in the body [5]), or to distinguish one particular chromophore from all of the other imaged ones without considering its dilution (e.g., determination of the level of vascularization and calculation of the concentration of oxygenated and deoxygenated blood, for oxygenation rate evaluation [6,7], cancer tissue evaluation [8], or imaging connectivity in brain [9]).

In photoacoustic images, each pixel can be interpreted as a mixture of pure chromophores, where the weights represent their average concentrations. The spectra of pure chromophores are called endmembers, and their weights are the abundance coefficients. Unmixing techniques for multispectral photoacoustic data are presented in this article, with the objective being to estimate the concentrations of the chromophores present in the imaged area. In the field of photoacoustic imaging, several unmixing methods have already been used to detect chromophores from the body [10] or contrast agents injected into tissues. For instance, principal component analysis (PCA) and independent component analysis were used in Reference [3]. However, the abundance matrices calculated by these two methods do not allow accurate estimation of concentrations of chromophores. In particular, these matrices can contain negative values that cannot be interpreted as concentrations even after scaling. This issue is one of the challenges addressed in this paper. Indeed, this study highlights experimental results as a proof of concept of the usefulness of photoacoustic imaging to evaluate chromophore concentration.

The concentration of an endmember should be equal to zero if the chromophore is absent in the considered pixel, and equal to one if it is a pure pixel. The endmember will lie in the interval (0,1) if the pixel is partly composed of the chromophore (i.e., diluted chromophore, or a mix of chromophores). The abundance matrix must then be subject to the following constraints: (1) each abundance coefficient must be in the range [0,1]; and (2) for a given pixel, the sum of all *K* abundance coefficients, which correspond to the *K* endmembers, must be less than 1 (i.e., diluted chromophore) or equal to 1 (i.e., pure chromophore, or a mixture of chromophores). These constraints have never been investigated in the field of photoacoustics, while they are usually encountered in remote sensing, where imaged areas might contain, for example, roads, buildings, rivers, or forests, which correspond to the chromophores to be unmixed. The abundances are, thus, related to the local concentrations of the chromophores in the scene. Spectral unmixing is also a widely explored field for various applications, like food safety [11,12], pharmaceutical process monitoring [13], and industrial and forensic applications [14,15]. However, unmixing of remote sensing data is the most closely related area to our multispectral photoacoustic data. As many efficient algorithms have been devised and analyzed over the last decade, we focus on this particular field in this study. The main challenge of this paper is then to show that photoacoustic imaging can benefit from these innovations, principally considering the constraints presented above, because of the similarities between these two fields. However, photoacoustic imaging contains less spectral bands than remote sensing and is subject to spectrally dependent light attenuation which makes the unmixing even more challenging. Moreover, the methods presented in this study are based on relative comparison between spectra. Even if PCA is also a relative method, as mentioned before, it does not allow proper evaluation of concentration, based on authors preliminary experiments. The study explores, then, other field to adapt unmixing methods that better suit the considered goal. As all spectra are impacted in the same way by the light absorption and ultrasound attenuation for each wavelength, and these methods allow us to avoid taking into account these parameters.

## 2. Unmixing of Photoacoustic Data

**Figure 1:**
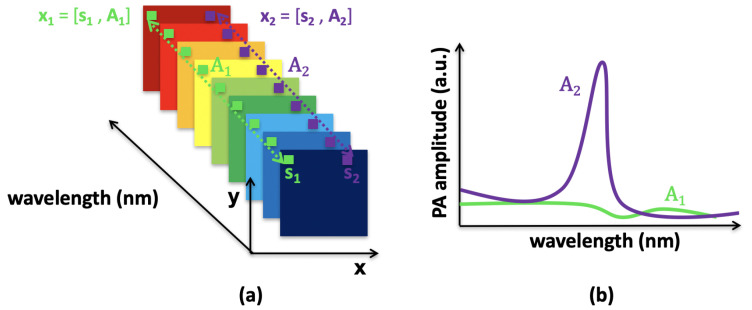
Multispectral photoacoustic image. (**a**) The signals are acquired at several wavelengths. Two pixels are identified: $x_{1}$ in green, and $x_{2}$ in purple. (**b**) Spectra of the pixels $x_{1}$ and $x_{2}$: **A**${}_{1}$ (in green) is a background pixel with low and flat photoacoustic amplitudes at all wavelengths, and **A**${}_{2}$ (in purple) is a pixel with significant photoacoustic amplitudes (a.u., arbitrary units).

A multispectral photoacoustic image consists of a two-dimensional area that is imaged at *L* wavelengths. Each pixel $x_{i}$ is characterized by its coordinates $s_{i}\in I\;N^{2}$ and is endowed with a spectrum $A_{i}\in I\;R^{L}$. The *N* samples, $x_{i}$, of the region of interest are expressed as follows:

(1) $$x_{i}=\left[s_{i},A_{i}\right]\in X,$$

with i∈{1,…,N} as the sample index; see Figure 1a. Before any other processing, the data need to be normalized over the range [0,255]. This allows the same range of values for data from different acquisition systems. Two types of pixels are illustrated in Figure 1: pixel **x**${}_{1}$ contains only noise, called the background, with low and flat photoacoustic amplitudes at all wavelengths (Figure 1b, green curve **A**${}_{1}$), and pixel **x**${}_{2}$ provides information on the optical absorbers, with a high photoacoustic signal (Figure 1b, purple curve **A**${}_{2}$).

### 2.1. Linear Mixing Model

The acquisition of photoacoustic data over a region of interest at several wavelengths provides a multispectral (i.e., three-dimensional) image, where each pixel is endowed with a spectrum. This spectrum is either pure, and is then considered as a single endmember, or mixed if it is composed of a mixture of endmembers. The linear mixing model (LMM) [16] considers that each mixed pixel is a convex combination of the spectra of the endmembers. More formally, this is defined as follows:

(2) $$\begin{array}{cc} A_{i} & =\sum_{k=1}^{K}u_{ki}E_{k}+g_{i},\;\;\forall \;i\in \left\{1,\ldots ,N\right\}, \end{array}$$

where $A_{i}\in I\;R^{L}$ is the spectrum at the *i*-th pixel, *L* is the number of wavelengths, *K* is the number of endmembers, $u_{ki}$ is the abundance of the *k*-th endmember in the *i*-th pixel, $E_{k}$ is the *L*-dimensional spectrum of the *k*-th endmember, and $g_{i}$ is a vector of zero-mean white noise that defines the sensor noise and the error of the model. All of the vectors are column vectors. Then, U is used for the matrix of abundances with the generic (i,j)-th entry $u_{ij}$. As abundances are relative contributions, they must be positive and their sum has to be equal to one:

(3) $$\left\{\begin{array}{c} u_{ki}\geq 0,\;\forall \;i\in \left\{1,\ldots ,N\right\},\;\forall k\in \left\{1,\ldots ,K\right\}\\ \sum_{k=1}^{K}u_{ki}=1,\;\forall \;i\in \left\{1,\ldots ,N\right\}. \end{array}\right.$$

The LMM is a simple but effective model that is widely used in remote sensing [16]. For medical applications, the LMM can be a powerful tool for estimation of concentration of chromophores. In this context, it is assumed that the observed spectra can either correspond to a fully concentrated chromophore or a diluted chromophore, or to a mixture of several chromophores. The original LMM with the constraints of Equation (3) does not consider dilution. It can, however, be extended to this scenario by relaxing the sum-to-one constraint, which can be performed by considering a zero endmember with the LMM, called a shadow endmember.

### 2.2. Unmixing Strategy

Data unmixing requires inversion of a mixing model that can be either linear or nonlinear, depending on the hypotheses. The study focus on unsupervised methods to be robust to the various photoacoustic systems that exist. Unsupervised algorithms have been devised in remote sensing to extract endmembers and to estimate the abundance matrix. Unsupervised methods, such as group lasso with unit sum and positivity constraints (GLUP) [17] and vertex component analysis (VCA) [18], can do both of these simultaneously, subject to constraints (3). Other unsupervised methods, such as N-FINDR [19], only extract the endmembers. A supervised unmixing algorithm is then required to calculate the abundances. The fully constrained least-square algorithm (FCLS) [20] usually provides satisfactory performance as long as the endmembers are accurately extracted. FCLS can be used with GLUP, VCA, and N-FINDR [17], or with any other strategy for extraction of the endmembers. In this study, we use FCLS to estimate the abundance matrices.

In the following, we use GLUP, VCA (that was already used in photoacoustic imaging in Reference [21]), and N-FINDR to estimate the endmembers in experimental multispectral photoacoustic data to evaluate the interest of remote sensing methods to process these data. We compare their performances with the spatio-spectral mean-shift (SSM-S) algorithm [22] devised by some of the present authors.

## 3. Method

### 3.1. Pre-Processing

The pre-processing introduced in this section aims to discriminate pixels of interest from the background. Among the possible strategies, background pixels can be determined by thresholding filtered data with a Sobel filter. The Sobel threshold $\lambda_{S}$ can be set as follows:

(4) $$\lambda_{S}=2*\sqrt{{\bar{\nabla }}_{S}},$$

where ${\bar{\nabla }}_{S}$ is the mean of the Sobel gradient magnitude [23]. For our application, only the edges detected by the Sobel filter were used to calculate ${\bar{\nabla }}_{S}$. Threshold $\lambda_{S}$ was applied to the sum over all of the wavelengths of the multispectral photoacoustic image, to design a mask where the background pixels were set to 0. The estimation methods introduced hereafter were only applied to pixels out of the background.

### 3.2. Endmember Extraction

#### 3.2.1. GLUP Algorithm

Group lasso with unit sum and positivity constraints assumes that the endmembers are not known but are present in the form of some pure (unmixed) pixels in the image [17]. Based on this assumption, the LMM (2) can be reformulated as follows:

(5) $$\begin{array}{cc} A_{i} & =\sum_{j=1}^{N}{u_{G}}_{ji}A_{j}+g_{i},\;\forall \;i\in \left\{1,\ldots ,N\right\}, \end{array}$$

where ${u_{G}}_{ji}$ is the abundance of $A_{j}$ in $A_{i}$. On the one hand, if $A_{j}$ is an endmember, the *j*-th row $U_{G_{j}}$ of matrix $U_{G}$ calculated with GLUP should have nonzero entries that represent the abundance map of $A_{i}$. On the other hand, if $A_{j}$ is a mixed pixel, $U_{G_{j}}$ should have all of its entries equal to zero. This means that $U_{G}$ should have N−K rows of zero, with the other *K* rows equal to the rows of U.

The premise in GLUP is that $U_{G}$ allows the identification of the endmembers in observation A through its nonzero rows. The resulting unmixing problem requires that $U_{G}$ only has a few rows different from zero, in addition to the non-negativity and sum-to-one constraints. This leads to the following convex optimization problem:

(6) $$\min_{U_{G}}\;\left(\frac{1}{2}\sum_{j=1}^{N}∥A-AU_{G}{∥}_{F}^{2}+\mu \sum_{j=1}^{N}∥U_{G_{j}}{∥}_{2}\right)$$

$$\begin{array}{cc} \mathrm{subject}\;\mathrm{to}: & \left\{\begin{array}{c} u_{G_{ji}}\geq 0,\;\forall \;i,j\in \left\{1,\ldots ,N\right\}\\ \sum_{j=1}^{N}{u_{G}}_{ji}=1,\;\forall \;i\in \left\{1,\ldots ,N\right\}, \end{array}\right. \end{array}$$

where μ>0 is a small regularization parameter that is set by the user, and $A\;=\;[A_{1},\ldots ,A_{N}]$ is the matrix of all pixels to unmix. The first term in Eqtuation (6) ensures that the observations match the model in Equation (5), and the second term is the group lasso regularizer that induces sparsity by possibly driving several rows of $U_{G}$ to zero [24]. The minimization problem has constraints to ensure that the abundances obey the positivity and the sum-to-one constraints. This optimization problem can be solved with a primal-dual method; see Reference [17] for details.

To conclude, GLUP allows the identification of the endmembers in A by identification of the nonzero rows in $U_{G}$. GLUP also provides the estimated abundances that correspond to the nonzero rows in the estimated matrix $U_{G}$. In this paper, similarly to Reference [17], we did not use the abundances estimated by GLUP. For a fair comparison with other algorithms, we re-estimated the abundances with the FCLS algorithm from the endmember spectra estimated using GLUP.

#### 3.2.2. VCA Algorithm

Vertex component analysis also assumes the presence of pure pixels in the data [18]. The driving principle of the VCA algorithm is to project the data onto a direction orthogonal to the subspace spanned by the endmembers already extracted. The new extracted endmember is the farthest signal in this projection domain. Considering this endmember, a new subspace is calculated, and the same procedure is iteratively performed until the preset number *K* of endmembers to extract is reached.

The first step of the VCA algorithm consists of determination of the initial subspace. Two methods are recommended, which depend on the signal-to-noise ratio. If the signal-to-noise ratio is greater than $\lambda_{V}$ in Equation (7), this first subspace is calculated using the singular value decomposition algorithm [25]. Otherwise, the subspace considered is constructed using principle component analysis. The threshold $\lambda_{V}$ is defined in Reference [18] as follows:

(7) $$\lambda_{V}=15+10\log_{10}\left(K\right).$$

Let us use $S_{k}$ to denote the subspace available at iteration *k*. A vector $v_{k}$ orthonormal to $S_{k}$ is calculated as follows:

(8) $$v_{k}=\frac{r_{k}-S_{k}S_{k}^{+}r_{k}}{∥r_{k}-S_{k}S_{k}^{+}r_{k}∥},$$

where $r_{z}$ is a zero-mean random vector, and $S_{z}^{+}$ is the pseudo inverse of $S_{k}$. The observed spectra A are then projected onto direction $v_{k}$:

(9) $$f_{k}=v_{k}^{⊤}A.$$

The largest entry of $f_{k}$ in absolute values allows the designation of the spectrum in A to be considered as a new endmember. This endmember is then added to the set of endmembers that have already been extracted, to define the subspace $S_{k+1}$ that is considered at the next iteration. The procedure is stopped when the number *K* of desired endmembers is reached. They are stored in matrix $E_{V}$, and the abundance matrix $U_{V}$ is calculated by projecting A onto $E_{V}$. For a fair comparison with other algorithms, we estimated the abundances with the FCLS algorithm from the endmember spectra $E_{V}$ estimated with VCA.

#### 3.2.3. N-FINDR Algorithm

N-FINDR also assumes that a pure pixel for each chromophore to unmix is present in the dataset [19,26]. The first step is the random generation of a set of *K* endmembers to produce the matrix $E_{0}$ of endmembers that are available at iteration 0. At each iteration *k*, the following volume is calculated:

(10) $$V_{k}=\frac{\left|\det(E_{k})\right|}{(K-1)!},$$

$$\begin{array}{cc} \mathrm{with} & E_{k}=\left[\begin{array}{ccc} 1 & \ldots & 1\\ E_{k}^{\left(1\right)} & \ldots & E_{k}^{\left(K\right)} \end{array}\right], \end{array}$$

where $E_{k}^{\left(p\right)}$ is the *p*-th endmember in the matrix of endmembers $E_{k}$ available at the *k*-th iteration. All of the pixels $A_{i}$ in A take place successively in $E_{k}$, where they are successively substituted to all possible columns *p*. Volume $V_{k+1}$ is updated as follows, for instance:

(11) $$V_{k+1}=\frac{\left|\det\left[\begin{array}{ccccc} 1 & \ldots & 1 & \ldots & 1\\ E_{k}^{\left(1\right)} & \ldots & A_{i} & \ldots & E_{k}^{\left(K\right)} \end{array}\right]\right|}{(K-1)!}.$$

If the new volume is greater than the previous one, $V_{k}$ and $E_{k}$ are updated with the new values. The procedure is stopped when the *N* pixels of the dataset have been tested. The resulting endmembers are stored in matrix $E_{N}$. No abundance coefficients are calculated with this method, and the FCLS can be used to this end.

#### 3.2.4. SSM-S Algorithm

Spatio-spectral mean-shift is a clustering method that was introduced by Reference [22] for chromophore discrimination in multispectral photoacoustic images. It is based on a spatio-spectral regularization, to cluster the pixels that are spatially and spectrally close.

Consider a pixel $x_{i}$. Its neighbors in spatial dimensions at a radial distance less than $R_{S}$ are first considered. These pixels $x_{j}=\left[s_{j},A_{j}\right]$ have to satisfy the following:

(12) $$\frac{1}{R_{S}}{∥s_{i}-s_{j}∥}_{2}\leq 1.$$

Assuming that spectra of the same chromophore are close, we also consider that two spectra can be from the same chromophore if their distance is less than $R_{\lambda }$; namely,

(13) $$\frac{1}{R_{\lambda }}{∥A_{i}-A_{j}∥}_{\infty }\leq 1.$$

Note that we found it interesting in Equation (13) to consider the infinite norm, to discriminate more effectively spectra that might differ only in narrow frequency bands.

The SSM-S only applies to pixels that satisfy both constraints of Equations (12) and (13). It consists of updating their locations and spectra as follows:

(14) $$x_{i}^{\left[t+1\right]}=\frac{\sum_{\begin{array}{c} j=1 \end{array}}^{N}g_{S}\left(s_{i}^{\left[t\right]};s_{j}^{\left[t\right]}\right)\cdot g_{\lambda }\left(A_{i}^{\left[t\right]};A_{j}^{\left[t\right]}\right)\cdot x_{j}^{\left[t\right]}}{\sum_{\begin{array}{c} j=1 \end{array}}^{N}g_{S}\left(s_{i}^{\left[t\right]};s_{j}^{\left[t\right]}\right)\cdot g_{\lambda }\left(A_{i}^{\left[t\right]};A_{j}^{\left[t\right]}\right)},$$

where $g_{S}\left(s_{i}^{\left[t\right]};s_{j}^{\left[t\right]}\right)=1$ and $g_{\lambda }\left(A_{i}^{\left[t\right]};A_{j}^{\left[t\right]}\right)=1$, if Equations (12) and (13) hold, respectively, and 0 otherwise. This iterative procedure is applied to all of the pixels $x_{i}$ until convergence; see Reference [22] for details. Unlike the GLUP, VCA, and N-FINDR algorithms, note that SSM-S uses spatial information in addition to spectral information. SSM-S is studied here because of this particular capacity which is not often taken into account to process multispectral photoacoustic data. Updating of Equation (14) leads to clusters of pixels, with each defined by a centroid. Each centroid can be considered as an endmember and stored in a matrix, which is denoted by $E_{S}$. As no abundance coefficients are calculated with this method, the FCLS can be used to this end.

Before concluding this section, we want to draw attention to the following: that, unlike the N-FINDR and SSM-S algorithms, GLUP and VCA converge to stationary points that can vary significantly depending on their initialization.

### 3.3. Abundances Estimation

The FCLS algorithm is widely used in remote sensing to estimate abundances, based on endmember knowledge. It considers the constraints of Equation (3) when solving the following optimization problem:

(15) $$\min_{U}\;\left(\frac{1}{2}\sum_{j=1}^{N}{∥A-UE∥}_{F}^{2}\right)\prime$$

$$\begin{array}{cc} \mathrm{with} & \left\{\begin{array}{c} u_{ji}\geq 0,\;\forall \;i,j\\ \sum_{j=1}^{N}u_{ji}=1,\;\forall \;i \end{array}\right. \end{array}$$

## 4. Materials

### 4.1. Acquisition System

**Figure 2:**
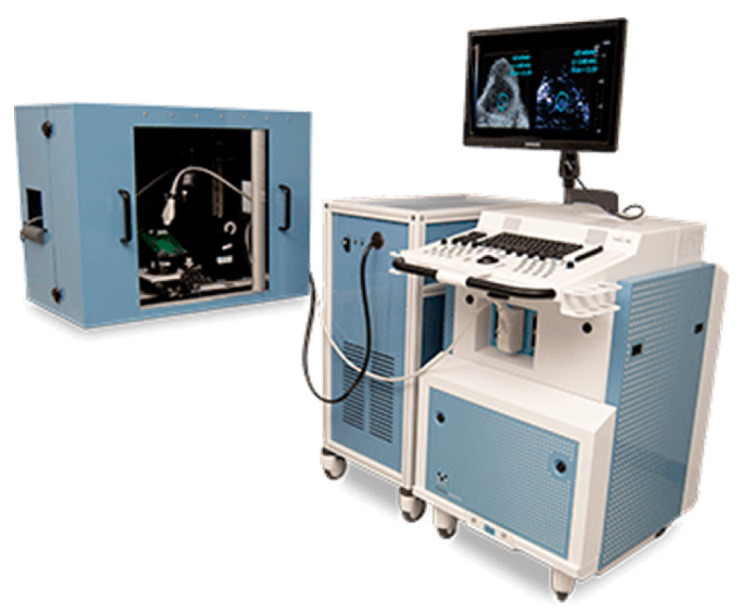
Commercial multispectral photoacoustic system: Vevo LAZR.

A commercial multispectral photoacoustic system (Vevo LAZR; Visualsonics, Fujifilm) was used for this study; see Figure 2. The optical source of this system is a Nd:YAG pulsed laser with a pulse duration of 5 ns and a 20 Hz repetition rate, which is coupled to an optical parametric oscillator to select the transmission wavelengths [27]. The wavelength can be set from 680 nm to 970 nm. The photoacoustic probe that was used (LZ400) is composed of 256 elements, which can acquire ultrasound signals in the frequency range from 18 MHz to 38 MHz. The imaging depth is approximately 1.5 cm and the imaged region of interest is around 1 cm. The images are reconstructed with a delay-and-sum algorithm. For every acquisition, the corresponding fluence is saved. For the acquisitions presented in this study, the fluence is varying of only 2% between the minimal and maximal fluences. As the variation is low and the proposed methods are based on unsupervised endmembers extraction with relative comparison between spectra, the images were not normalized by the fluence.

**Figure 3:**
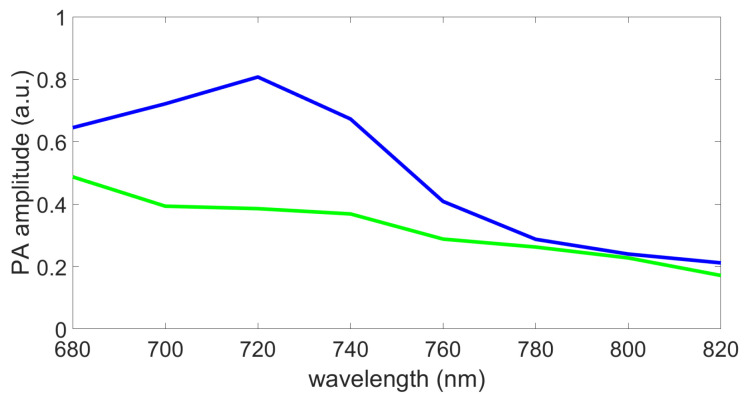
Spectra, measured with a Perkin Elmer Lambda900 spectrometer for the study [28], of the blue and green inks used to make the phantoms (a.u., arbitrary units).

The data were acquired using the full range of available wavelengths (680 nm,970 nm) with 1 nm steps, which required less than 1 min of acquisition time. The time to switch from one wavelength to the other and to acquire the corresponding image is around 0.2 s. Usually, in multispectral photoacoustic imaging, from 5 to 10 wavelengths are used because the information collected is then sufficient to discriminate or quantify biological chromophores with an acceptable processing time. To get closer to this framework, only eight of the acquired wavelengths were selected to construct our dataset, from 680 nm to 820 nm, with 20 nm steps. These wavelengths were chosen because the used optical absorbers (blue and green inks) can be discriminated in this range (see Figure 3).

The algorithms were computed with MATLAB (The MathWorks Inc., Natick, MA, USA) on a computer with a 16-GB memory and an Intel Core i7-5500U CPU at 2.40 GHz.

### 4.2. Imaged Phantoms

**Figure 4:**
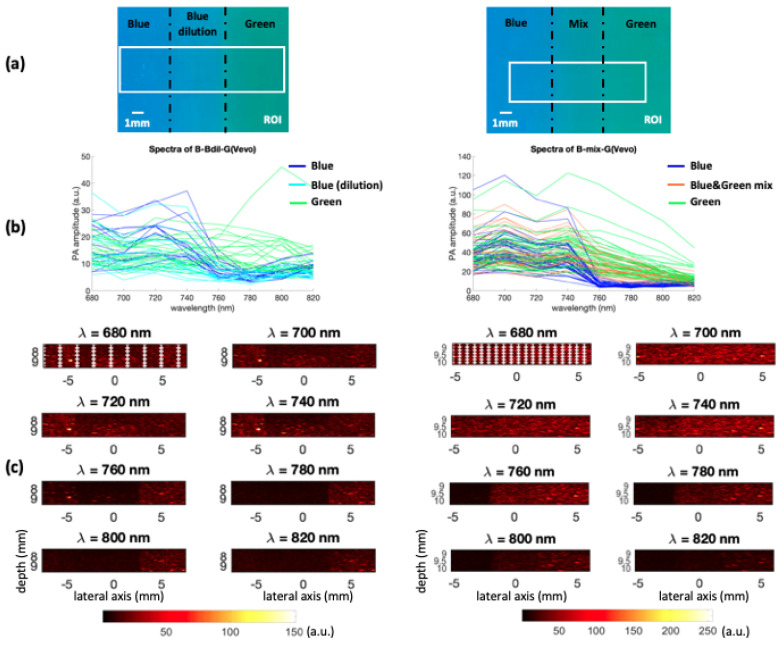
(**a**) Left: Blue/0.53 blue dilution/green colored phantom. Right: Blue/0.42 blue & 0.67 green mix/green colored phantom. (**b**) Spectra of each region of interest. Each spectrum corresponds to a specific pixel. These pixels are highlighted on the images (**c**) at λ=680 nm. (a.u., arbitrary units) (**c**) Left: The ***B-Bdil-G(Vevo)*** dataset (mean value over all 8 wavelengths: 12.05 a.u.). Right: The ***B-mix-G(Vevo)*** dataset (mean value over all 8 wavelengths: 26.72 a.u.). The image axes are in millimeters.

The homemade colored phantoms used to construct the dataset were composed of three different 4% colored agar parts. The ones on the left side in Figure 4a were prepared using only blue ink (400 g water, 16 g agar, 380 μL ink), and the ones on the right with only green ink (400 g water, 16 g agar, 950 μL ink). Both of these are considered as relative ink concentrations equal to 1 (i.e., pure chromophores). The blue and green ink quantities were not equal because both of these inks do not show the same maximum photoacoustic signal amplitude. Based on a previous study [28], the ink quantities were chosen to obtain the same maximum photoacoustic signal amplitude, on the range from 400 nm to 1200 nm, for both of these pure chromophores. The used blue and green inks spectra, measured with a Perkin Elmer Lambda900 spectrometer for the study [28], are shown in Figure 3, on the wavelength range of interest for the present study.

In Figure 4b, the sample of shown spectra seems to highlight that the fully concentrated blue and green parts do not exhibit the same, even approximately, spectral intensities for both phantoms. However, this is only due to the spectra shown here. Nevertheless, the Figure shows that the shape of blue or green spectra are quite similar which is the property used by the proposed methods in this study.

#### 4.2.1. Chromophore Dilution

The central part of the left phantom in Figure 4a is composed of a 0.53 dilution of the blue ink relative to the pure concentration indicated above. Therefore, here 100 g water, 4 g agar, and 50 μL blue ink were used. This phantom is referred to as B-Bdil-G(Vevo) in the following.

#### 4.2.2. Mixing of the Chromophores

The central part of the second phantom, shown in Figure 4a-right, is a mix of the blue and green inks (50 g water, 2 g agar, 20 μL blue ink, 80 μL green ink). This means that the mix corresponds to a blue concentration of 0.42 and a green concentration of 0.67, relative to the pure concentrations indicated above. This phantom will be referred to as ***B-mix-G(Vevo)*** in the following.

### 4.3. Performance Evaluation on Phantoms

The performances of the GLUP/FCLS, VCA/FCLS, N-FINDR/FCLS, and SSM-S/FCLS strategies were evaluated on the phantoms described in Section 4.2. This means that FCLS was used to estimate the concentrations of the endmembers extracted beforehand with the other methods presented in Section 3.2. The resulting abundance maps were compared to the ground truth.

**Figure 5:**
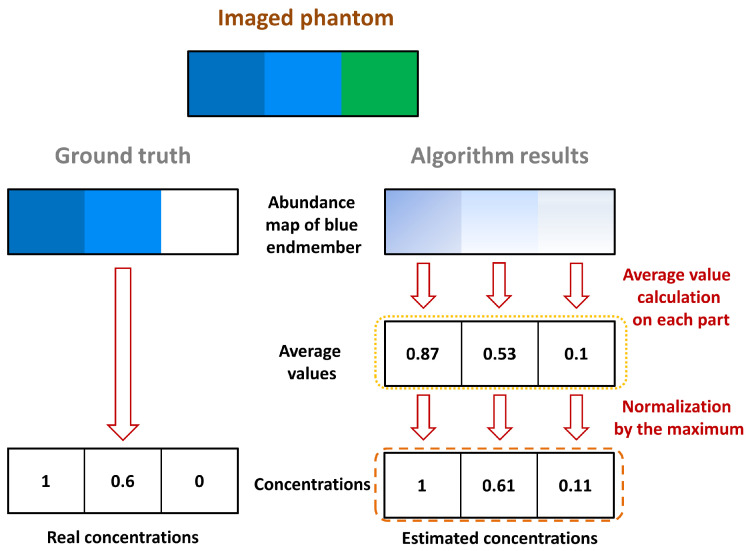
Performance validation procedure on an illustrative synthetic phantom. The validation here is for the estimation of the blue concentration.

For each abundance map of each endmember, a mean concentration was calculated for each part of the imaged region, as illustrated in Figure 5. The illustrative phantom considered in Figure 5 is composed of a pure blue chromophore, a dilution of this chromophore, and a pure green chromophore (from left to right). Considering the abundances related to the blue endmember, three average values were calculated: one for each part, as circled by the yellow dotted line in Figure 5. As we assume that the pure blue chromophore, which corresponds to the endmember of interest, is present in the image, the mean values were normalized by their maximum, as circled by the orange dashed line in Figure 5. Where the data would be perfectly unmixed, the maximum value should correspond to the pure blue part of the phantom (i.e., the left part), which is then set to 1 after normalization. The other normalized values should correspond to the relative concentrations of the pure chromophore, i.e., the relative concentration of blue in the illustrative example in Figure 5.

This calculation was performed to estimate the abundance maps provided by the different unmixing strategies. Indeed, by considering the light absorption and the diffusion in tissue, each pixel of similar region exhibit similar spectra shape tendency (see Figure 4b). For each pixel, a particular concentration is then evaluated. The normalized mean values were compared with the ground truth to evaluate the performances of the algorithms on each whole part.

### 4.4. In Vivo Data Acquisitions

**Figure 6:**
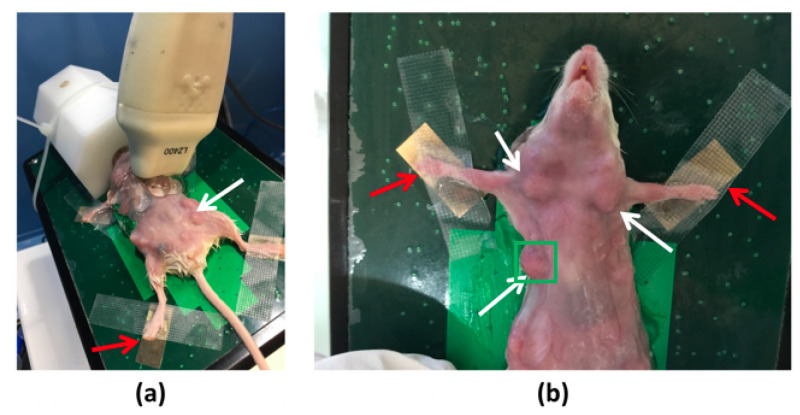
Mouse with breast tumors in the Vevo LAZR system. White and red arrows indicated the tumors and heartbeat detectors, respectively. In (**a**) the anesthesia mask can be seen (white block). The green square in (**b**) highlights the imaged region-of-interest.

Images were acquired with the Vevo LAZR system. For imaging the mice tumors, the BALBc MMTV-Neu transgenic mouse model was used, which expresses the Her-2/neu V664E oncogene [29]. This model develops spontaneous mammary tumors that are visible with the photoacoustic system after 17 weeks (between 5 mm${}^{3}$ and 10 mm${}^{3}$ in size). Palpable tumors are detected around 20 weeks old; see Figure 6, where the white arrows indicate some of the tumors, and the green square denote the imaged one. A 20-week-old mouse was used for the imaging, where the region of interest was shaved the day before using a commercial hair remover cream (Veet, Cream hair remover; Reckitt Benckiser, UK) to avoid any imaging noise coming from the mouse hair. The mouse was anesthetized with a mix of 3.5% isoflurane and 96.5% oxygen before the photoacoustic acquisitions, using an anesthesia mask. During the imaging procedure, the mouse was maintained asleep through inhalation of a mix that contained 1.5% isoflurane (Figure 6a), to minimize movements during the acquisitions at the different wavelengths. Heartbeat and breathing were monitored by several devices connected to the mouse paws (Figure 6b, red arrows) and the anesthesia mask, respectively. This allowed monitoring of the health of the mouse and the acquisition of images at the same position. In this way, heartbeat and breathing motions were avoided as much as possible. The mice were maintained in the specific pathogen free animal facility at the Cancer Research Center of Lyon, at Center Léon Bérard. All of the mice experiments were performed in accordance with the animal care guidelines of the European Union and were validated by the local Animal Ethics Evaluation Committee and the French Ministry of Higher Education and Research (C2EA-15 and 02296.02).

The dataset used to test this strategy was composed of 15 images that were acquired from 680 nm to 960 nm, in 20 nm steps. The results were compared to the Vevo LAZR sO${}_{2}$ map. To this end, acquisitions on the same tumor were also performed using the Vevo LAZR Oxy-Hemo mode.

### 4.5. sO${}_{2}$ Calculation with Vevo LAZR Oxy-Hemo Mode

The Oxy-Hemo mode acquires two photoacoustic images, at 750 nm and 850 nm. With both of these images, the concentrations of deoxygenated hemoglobin (Hb) and oxygenated hemoglobin (HbO${}_{2}$) were measured, which are expressed as [Hb] and [HbO${}_{2}$], respectively [30]. The oxygen saturation was then calculated as follows:

(16) $${\mathrm{sO}}_{2}=\frac{{[\mathrm{HbO}}_{2}]}{\left[\mathrm{Hb}\right]+{[\mathrm{HbO}}_{2}]}.$$

This ratio is classically used in biomedical applications to provide oxygenation information [31,32]. Both of the concentration maps were saved, as well as the Vevo LAZR sO${}_{2}$ displayed map, for further comparisons. The sO${}_{2}$ acquisition is conducted just after the acquisitions on the whole range of wavelength, allowing then a fair comparison of the estimated concentrations.

## 5. Results

As indicated in Section 3.2, the N-FINDR and SSM-S algorithms converge to a single set of estimated endmembers, regardless of their initialization. On the contrary, the GLUP and VCA methods can converge to different sets of endmembers, which depend on the initialization. For the acquisitions with the phantoms, the results presented for GLUP and VCA are the best ones that were achieved. For the preliminary in vivo results, the sets of endmembers obtained with the different techniques are presented and discussed.

### 5.1. Chromophore Dilution

**Figure 7:**
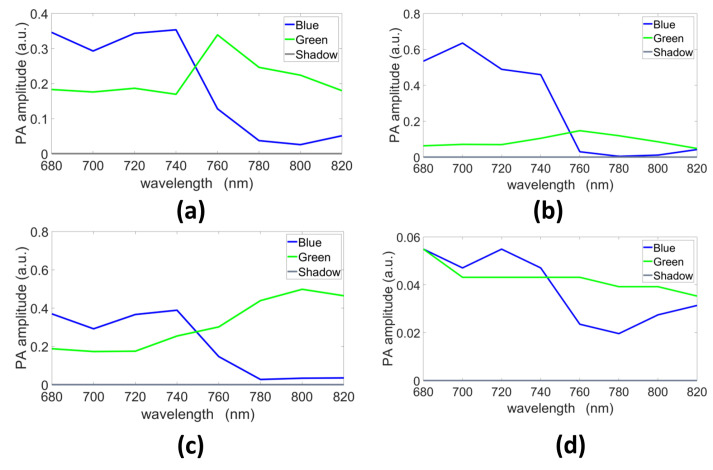
Endmembers estimated with (**a**) GLUP, (**b**) VCA, (**c**) N-FINDR, and (**d**) SSM-S (a.u., arbitrary units).

The endmembers estimated with GLUP, VCA, N-FINDR, and SSM-S are presented in Figure 7a–d, respectively. They allow us to discriminate the pure blue and green chromophores even if they do not exactly fit the reference spectra measured with a spectrometer (Figure 3). Indeed, as already mentioned, light absorption and diffusion in tissue impact the pure chromophore spectrum. The shape tendency is, however, kept. The shadow endmember is also plotted in each panel.

**Figure 8:**
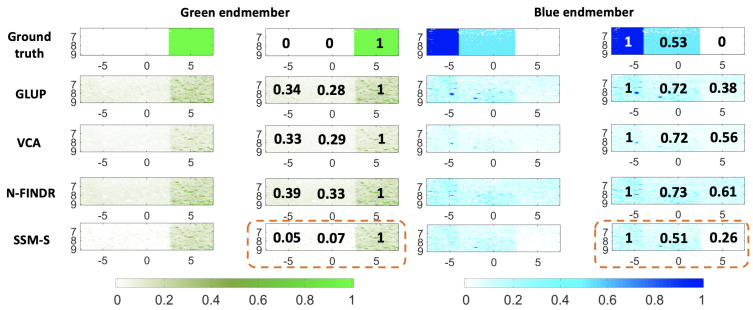
Unmixing method results for the ***B-Bdil-G(Vevo)*** dataset with the algorithms indicated, combined with FCLS. Left columns: green endmember and unmixing results summary. Right columns: blue endmember and unmixing results summary. The values in black are the average normalized values as explain in Figure 5. The ground truth is shown in both cases, in the first row. The image axes are in millimeters.

The abundance maps and the estimated mean concentrations are presented in Figure 8, for both the endmembers. All of these methods localize both of the pure chromophores well. The green abundance maps clearly highlight the green part, i.e., that on the right. The SSM-S achieves the best performance, as it leads to small green abundances for both parts of the blue phantom. Indeed, the mean concentrations are 0.05 and 0.07 for the blue and the diluted blue parts, respectively. The blue abundance maps are, nevertheless, also characterized by relatively large values in the green part. The SSM-S blue abundance map shows the smallest value of 0.26, and estimates a dilution value of 0.51, as compared to 0.53 for the ground truth. In conclusion, for this dataset, SSM-S/FCLS achieves the best performance.

### 5.2. Mixing of the Chromophores

**Figure 9:**
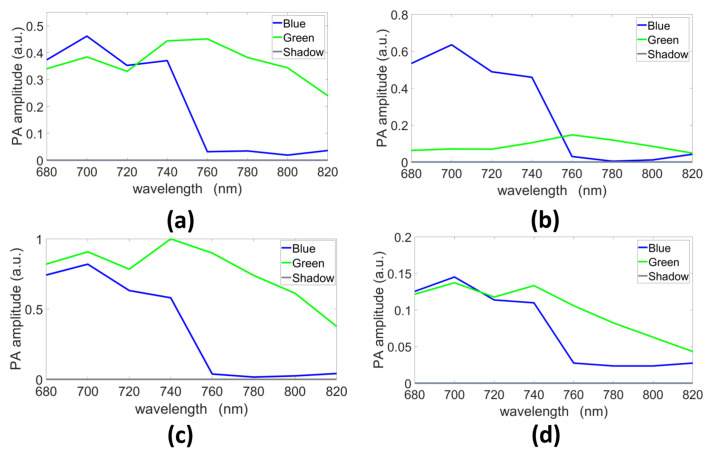
Endmembers extracted with (**a**) GLUP, (**b**) VCA, (**c**) N-FINDR, and (**d**) SSM-S (a.u., arbitrary units).

The unmixing methods were also tested with mixed chromophores using the ***B-mix-G(Vevo)*** dataset. The endmembers extracted by the different methods are presented in Figure 9. The extracted spectra differ from one strategy to another, but all blue spectra have similar evolutions, which matches the ground truth multispectral characteristics, in term of curve evolution, of this blue ink (Figure 3). The shadow endmembers are also plotted.

**Figure 10:**
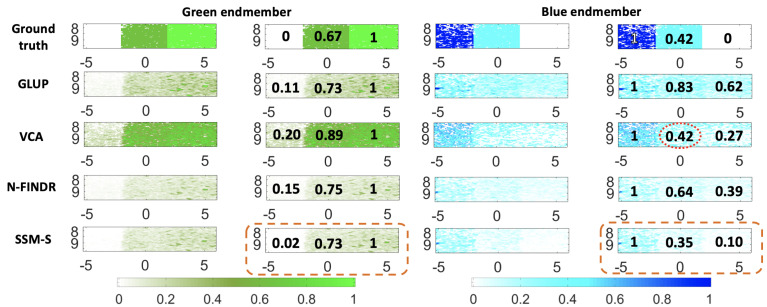
Unmixing method results for the ***B-mix-G(Vevo)*** dataset with the algorithms indicated, combined with FCLS. Left columns: green endmember and unmixing results summary. Right columns: blue endmember and unmixing results summary. The values in black are the average normalized values as explain in Figure 5. The ground truth is shown in both cases, in the first row. The image axes are in millimeters.

The abundance maps and the estimated mean concentrations are presented in Figure 10, for both of the endmembers. The blue abundance maps calculated with the endmembers extracted by GLUP and VCA localize the pure blue chromophore part (left) well, while the pure green part is more difficult to delimit. Indeed, the abundance values are similar in the central and right parts. For the N-FINDR and SSM-S blue abundance maps, the three parts are more visible. On the SSM-S abundance maps, the green part is characterized by a low concentration of 0.10, which is close to the ground truth value. For the green abundance maps, all of the methods allow discrimination of the blue part (left) and the mixed part (center). The VCA abundance map does not allow for clear discrimination of the mixed (central) and green (right) parts, while this is possible with the other methods.

For the estimated mean concentrations in Figure 10, the first result to be noted is the blue concentration of the mixed chromophore estimated using VCA, which is equal to the ground truth of 0.42, as circled by the red dashed line. Nevertheless, the other concentrations estimated with VCA are larger than the ground truth concentrations: 0.89 for the green in the mixed chromophore instead of 0.67, 0.27 for the blue in the pure green chromophore instead of 0, and 0.20 for the green in the pure blue chromophore instead of 0. Consider now the performance of the SSM-S/FCLS, as circled with the orange dashed line in Figure 10. The estimated concentrations in the mix of chromophores are 0.35 for the blue and 0.73 for the green, instead of 0.42 and 0.67 for the ground truth, respectively. This corresponds to an error of less than 7%, which is already an interesting result in terms of the targeting of biological applications. Note that the estimated abundances where the ground truth concentration equals 0 were estimated at values less than 0.1 by SSM-S, which is a satisfying performance compared to the other methods. As a conclusion, for this dataset, SSM-S/FCLS achieves the best performance.

### 5.3. Preliminary In Vivo Results

The analyzed strategies were used on biological tissues to calculate the oxygen saturation rate (sO${}_{2}$). This measurement is of great interest for various medical applications, such as the follow-up of tumors and the evaluation of tissue aging [33].

**Figure 11:**
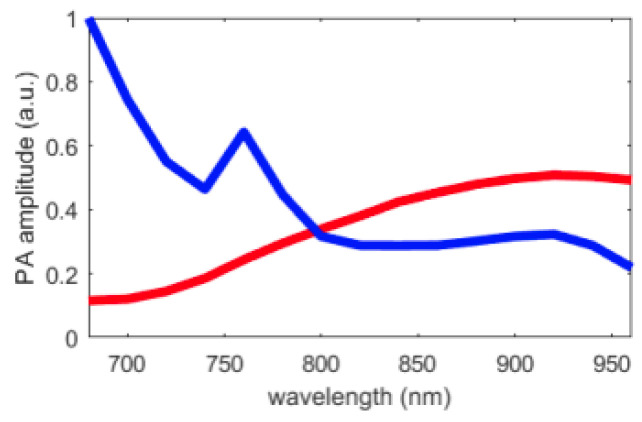
Theoretical Hb and HbO${}_{2}$ spectra (mean values are: 0.43 and 0.35, respectively).

**Figure 12:**
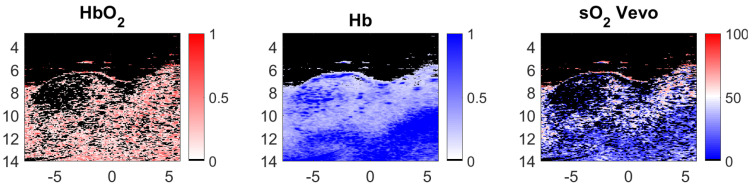
Oxygenated hemoglobin (HbO${}_{2}$) and deoxygenated hemoglobin (Hb) concentration maps and sO${}_{2}$ map (in %) calculated with the Vevo LAZR concentration maps and Equation (16), from left to right, respectively. The image axes are in millimeters.

The pre-processing steps described in Section 3.1 were followed. Then, the endmember estimation methods were applied to extract two endmembers, which corresponded to the Hb and HbO${}_{2}$ chromophores (the theoretical Hb and HbO${}_{2}$ spectra are shown Figure 11). Finally, the abundance maps of both of these endogeneous chromophores were estimated using the FCLS algorithm. The sO${}_{2}$ at each pixel was determined using the resulting abundance maps and Equation (16). These results were compared to the one provided by the Vevo LAZR. Indeed, the Vevo LAZR can provide abundance maps of the Hb and HbO${}_{2}$ chromophores. We used these abundance maps with Equation (16) to calculate the oxygen saturation rates; see Figure 12. The authors are aware that Vevo LAZR Oxy-Hemo mode is not the real ground truth. However, this scanner represents a significant reference since it is typically used for sO${}_{2}$ measurements in photoacoustic imaging.

**Figure 13:**
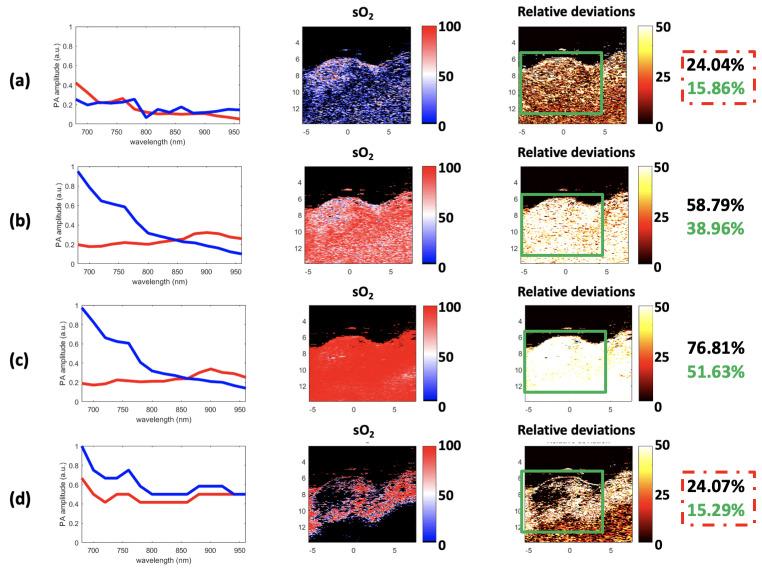
Results for (**a**) GLUP, (**b**) VCA, (**c**) N-FINDR, and (**d**) SSM-S. The extracted endmembers are shown (left). The mean values of the endmembers are: 0.17, 0.39, 0.41, and 0.61 for Hb (in blue) and 0.16, 0.24, 0.24, and 0.48 for HbO${}_{2}$ (in red), respectively for GLUP, VCA, N-FINDR, and SSM-S. The sO${}_{2}$ maps estimated using the FCLS with the extracted endmembers and Equation (16) are highlighted (center). The relative deviation maps between these sO${}_{2}$ maps and the Vevo LAZR map in Figure 12 are presented (right). The mean relative deviations for the whole image (black) and limited to the tumor (green) are given on the far right. The minimal relative deviations are circled in red. The image axes are in millimeters.

Figure 13, left column, shows the endmember spectra estimated with GLUP, VCA, N-FINDR, and SSM-S. Recall that the GLUP and VCA algorithms can converge to different sets of endmembers, depending on their initialization. The GLUP and VCA spectra represented in Figure 13 are those that then lead to the best sO${}_{2}$ maps compared to the Vevo LAZR map, which are considered as the relative ground truth. FCLS was used with all of these endmember spectra, to estimate the abundance maps of Hb and HbO${}_{2}$. The sO${}_{2}$ maps were then calculated using Equation (16); see Figure 13, center column. With this strategy, it appears that many pixels have strong sO${}_{2}$ values, when VCA and N-FINDR algorithms are used. In addition, the endmembers extracted with both these algorithms are really similar, but not exactly the same, even if it is quite hard to see their differences on Figure 13. However, the sO${}_{2}$ maps calculated with this strategy are different, which highlights the endmembers differences.

To characterize the performances of these methods, the relative deviations between each estimated sO${}_{2}$ map (see Figure 13, center column) and the Vevo LAZR map calculated with Equation (16) (see Figure 12, right column) were computed. This relative deviation defines the absolute difference between the Vevo LAZR sO${}_{2}$ values and each of the estimated sO${}_{2}$ values, for each pixel. This is provided in %, as the sO${}_{2}$ values are also in %. The relative deviation maps are presented in Figure 13, right column. Note that skin, tumor, and other biological tissues are present in the imaged area. The mean relative deviation was first calculated for all of these tissues. However, as the study was focused on the tumor only, the mean relative deviation was also calculated for the restricted regions delimited by the green rectangles in Figure 13. These relative deviations for all of these tissues and just for the tumor are in the black and green characters on the right in Figure 13, respectively. For both regions, the best estimations were reached with GLUP and SSM-S, which give similar relative deviations: 24.04% and 15.86% for GLUP, and 24.07% and 15.29% for SSM-S.

## 6. Discussion

This study experimentally evaluates the interest of using multispectral photoacoustic imaging to estimate chromophore concentration. To tackle such objectives, several methods coming from the remote sensing field have been tested to evaluate their potential performances in the photoacoustic domain. Principally, the constraints of Equation (3) can benefit also for chromophore concentration evaluation purpose. The interests and disadvantages of each method, highlighted by the in vivo proof of concept to evaluate sO${}_{2}$, are here discussed.

**Figure 14:**
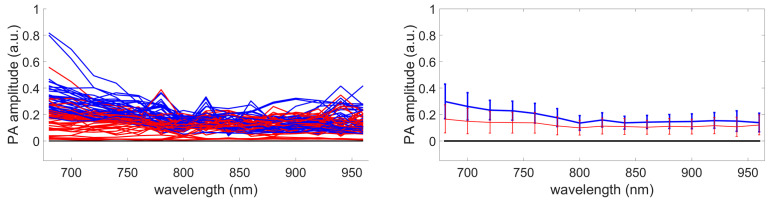
Endmembers extracted with GLUP. **Left** column: sets of extracted endmembers. **Right** column: mean spectra and standard deviations.

**Figure 15:**
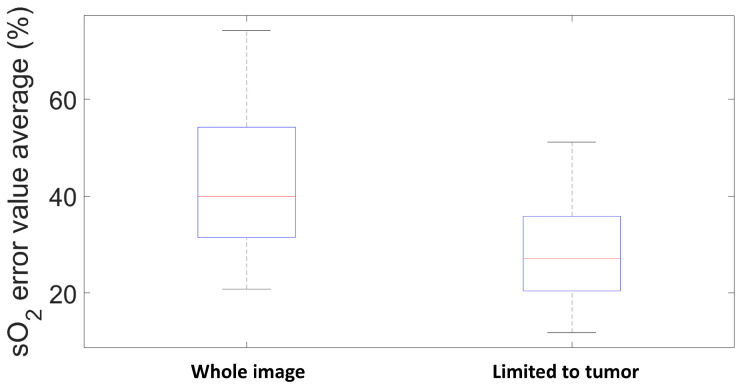
Mean relative deviations on sO${}_{2}$ maps estimated with GLUP over 200 runs.

**Figure 16:**
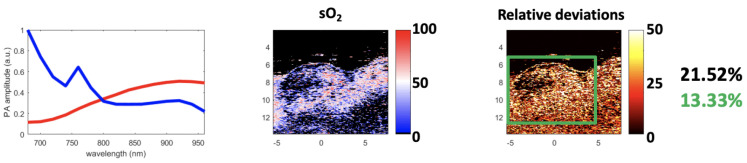
Results using the theoretical absorbance spectra of Hb and HbO${}_{2}$. (**a**) The theoretical spectra (mean values of Hb and HbO${}_{2}$ are: 0.43 and 0.35, respectively), (**b**) sO${}_{2}$ map estimated using the FCLS with the theoretical spectra and Equation (16), and (**c**) the relative deviation maps between the sO${}_{2}$ map and the Vevo LAZR map in Figure 12. The image axes are in mm.

First, the variabilities of GLUP and SSM-S have to be analyzed. As already mentioned, GLUP and VCA can converge to different sets of endmembers, which depend on their initialization. Here, we focus only on GLUP as VCA, regardless, gives inaccurate sO${}_{2}$ values. Figure 14 and Figure 15 illustrate this phenomenon with the results from 200 runs for the GLUP method. If GLUP can lead to small relative deviations, it should be noted that only 17 runs provided mean relative deviations less than 25%. In contrast, 59 runs provided mean errors greater than 50%. It is, however, interesting to note that the endmembers extracted with VCA and N-FINDR have the closest spectra to the theoretical ones [1]; see Figure 16a. This observation should be considered with caution though, as the theoretical spectra shown do not take into account the characteristics of the acquisition system, which might modify the acquired spectra. As well as for the inks study, light absorption and diffusion in tissue also impact the pure chromophore spectrum. This could explain the bad results given by these two methods even if the extracted spectra seem the similar ones.

Finally, considering its robustness, SSM-S is an interesting solution, as it converges to a unique solution while achieving a small relative deviation. However, it is important to note that the endmembers extracted with SSM-S do not look like the theoretical ones; see Figure 16a. As mentioned earlier, this could be due to the characteristics of the acquisition system and the light absorption and diffusion in tissue. In addition, in tumor tissues, pure Hb and HbO${}_{2}$ are not the most frequently present chromophores. The Vevo LAZR sO${}_{2}$ values (Figure 12) confirm this observation as they are mainly comprised between 25% and 75%, and then correspond to a mix of pure chromophores. This means that the endmembers that were actually extracted probably represent mixed chromophores instead of the pure Hb and HbO${}_{2}$. Regardless, the results obtained with the SSM-S/FCLS strategy are very encouraging in allowing accurate measurements of sO${}_{2}$ with photoacoustic imaging.

Table 1 summarizes all of the results, the abundances are calculated with the FCLS algorithm, and the mentioned methods are the ones used to extract the endmembers. This table shows that the minimum mean relative deviation was obtained with the theoretical spectra, but the mean relative deviations remain large, as these spectra do not take the characteristics of the acquisition system into account as well as the light absorption and diffusion in tissue. Nevertheless, this result is interesting, as it is independent of the Vevo LAZR Oxy-Hemo mode, and it provides a convenient basis for comparing results. In this sense, given the proximity to the performance obtained with SSM-S/FCLS, these complementary experiments confirm the potential of this strategy to quantify photoacoustic in vivo data. GLUP/FCLS also have similar performance but only for some runs, as mentioned before. This strategy could then be more studied and compared to SSM-S/FCLS on various cases.

However, the proposed strategies of hyperspectral imaging suffer from spectral coulouring effect [34,35]. Indeed, in the proposed strategies, the endmembers are evaluated without taking into account any fluence evolution through the sample. Such effect has demonstrated to bias the sO${}_{2}$ estimation and would required deeper study to be conducted. However, in the context of this work and its comparison to the sO${}_{2}$ evaluation of the Vevo LAZR, we did not evaluate its impact.

These results are encouraging to be used for various clinical applications requiring chromophore quantification. Indeed, the evaluation of chromophore concentrations is of major interest for various applications, like concentration of contrast agent in the body [5], calculation of the concentration of oxygenated and deoxygenated blood for oxygenation rate evaluation [6,7], or cancer tissue evaluation [8]. The endmembers extraction could be either automatic or manually selected by the user coupled with a fast inverse problem (FCLS).

## 7. Conclusions and Perspectives

Several methods for characterization of multispectral photoacoustic data have been considered, with the aim to estimate chromophore concentrations. These strategies mainly consist of two steps: (1) an endmember extraction step, to estimate the spectrum of each pure chromophore to be quantified; and (2) an abundance calculation step, to evaluate their concentrations. For the first step, different algorithms were compared (GLUP, VCA, N-FINDR, and SSM-S). The second step was performed using the FCLS algorithm.

For nonbiological tissues and using the Vevo LAZR acquisition system, we demonstrated that the SSM-S/FCLS procedure provides accurate estimates of chromophore concentrations for dilutions and mixtures. The positivity and sum-to-one constraints imposed by the FCLS, and coming from the remote sensing field, are interesting to take into account for chromophore concentration evalution. In some cases, the sum-to-one constraint is, however, questionable. It is a strong assumption, particularly when dilutions are imaged. To relax this constraint, a shadow endmember was introduced in this study, among the other possible strategies.

The methods were tested on a mouse tumor, where the sO${}_{2}$ calculation was performed and compared to the sO${}_{2}$ values calculated by the Vevo LAZR Oxy-Hemo mode. GLUP and SSM-S provided equivalent optimal performance to extract endmembers. GLUP algorithm should, however, be used with caution because of its variability. Nonetheless, it again highlights the interest of the constraints of Equation (3) in the photoacoustic field for chromophore concentration evaluation purpose. The SSM-S algorithm, which converges to a unique solution, appears to be more appropriate for endmember extraction, but further study on various cases should be done to quantify the performances of SSM-S/FCLS and GLUP/FCLS on various cases.

The endmember extraction and sum-to-one constraint relaxation still needs to be studied in more detail, but these primary results with biological tissues are encouraging. Moreover, the ground truth of the oxygenation rate in the imaged tumor was not known. It is, thus, difficult to know exactly which method is the most accurate to estimate the sO${}_{2}$ values. In addition, considering the presence of only Hb and HbO${}_{2}$ in the imaged area is a strong hypothesis. The water and other tissue absorbances should probably be taken into account. Further, other wavelengths can be tested to characterize their influence on the results. In the future, these experiments should be correlated with blood extraction and sO${}_{2}$ evaluation using blood measurements to have the real ground truth.

## Figures and Tables

**Table 1:** Summary of the average sO${}_{2}$ relative deviation values. The abundances are estimated using the FCLS algorithm.

Endmember Extraction	Whole Image	Limited to Tumor
GLUP	24.04%	15.86%
VCA	58.79%	38.96%
N-FINDR	76.81%	51.63%
SSM-S	24.07%	15.29%
Theoretical spectra	**21.52%**	**13.33%**

## Data Availability

The data presented in this study are available on request from the corresponding author.

## Abbreviations

The following abbreviations are used in this manuscript:

FCLS	Fully Constrained Least-Square
GLUP	Group Lasso with Unit sum and Positivity constraints
PCA	Principal Component Analysis
SSM-S	Spatio-Spectral Mean-Shift
VCA	Vertex Component Analysis

## References

[1] Beard P. (2011). Biomedical photoacoustic imaging. Interface Focus.

[2] van Veen R.L.P., Sterenborg H.J.C.M., Pifferi A., Torricelli A., Cubeddu R. Determination of VIS-NIR absorption coefficients of mammalian fat, with time- and spatially resolved diffuse reflectance and transmission spectroscopy. Proceedings of the Biomedical Topical Meeting.

[3] Glatz J., Deliolanis N.C., Buehler A., Razansky D., Ntziachristos V. (2011). Blind source unmixing in multi-spectral optoacoustic tomography. Opt. Express.

[4] Razansky D., Vinegoni C., Ntziachristos V. (2007). Multispectral photoacoustic imaging of fluorochromes in small animals. Opt. Lett..

[5] Mienkina M.P., Friedrich C.S., Hensel K., Gerhardt N.C., Hofmann M.R., Schmitz G. (2009). Evaluation of Ferucarbotran (Resovist) as a Photoacoustic Contrast Agent. Biomed. Eng..

[6] Mercep E., Dean-Ben X.L., Razansky D. (2017). Combined pulse-echo ultrasound and multispectral optoacoustic tomography with a multi-segment detector array. IEEE Trans. Med Imaging.

[7] Singh M.K.A., Sato N., Ichihashi F., Sankai Y. In vivo demonstration of real-time oxygen saturation imaging using a portable and affordable LED-based multispectral photoacoustic and ultrasound imaging system. Proceedings of the SPIE 10878, Photons Plus Ultrasound: Imaging and Sensing 2019.

[8] Jnawali K., Chinni B., Dogra V., Rao N. (2020). Automatic cancer tissue detection using multispectral photoacoustic imaging. Int. J. Comput. Assist. Radiol. Surg..

[9] Nasiriavanaki M., Xia J., Wan H., Bauer A.Q., Culver J.P., Wang L.V. (2014). High-resolution photoacoustic tomography of resting-state functional connectivity in the mouse brain. Proc. Natl. Acad. Sci. USA.

[10] Tzoumas S., Deliolanis N.C., Morscher S., Ntziachristos V. (2014). Unmixing Molecular Agents From Absorbing Tissue in Multispectral Optoacoustic Tomography. IEEE Trans. Med Imaging.

[11] Gowen A., O’Donnell C., Cullen P., Downey G., Frias J. (2007). Hyperspectral imaging—An emerging process analytical tool for food quality and safety control. Trends Food Sci. Technol..

[12] Bermana M., Connorb P., Whitbournb L., Cowardb D., Osbornec B., Southanc M. (2007). Classification of Sound and Stained Wheat Grains Using Visible and near Infrared Hyperspectral Image Analysis. J. Near Infrared Spectrosc..

[13] Gendrin C., Roggo Y., Collet C. (2008). Pharmaceutical applications of vibrational chemical imaging and chemometrics: A review. J. Pharm. Biomed. Anal..

[14] Picon A., Ghita O., Whelan P.F., Iriondo P.M. (2009). Fuzzy Spectral and Spatial Feature Integration for Classification of Nonferrous Materials in Hyperspectral Data. IEEE Trans. Ind. Inform..

[15] Brewer L.N., Ohlhausen J.A., Kotula P.G., Michael J.R. (2008). Forensic analysis of bioagents by X-ray and TOF-SIMS hyperspectral imaging. Forensic Sci. Int..

[16] Keshava N., Mustard J.F. (2002). Spectral unmixing. IEEE Signal Process. Mag..

[17] Ammanouil R., Ferrari A., Richard C., Mary D. (2014). Blind and fully constrained unmixing of hyperspectral images. IEEE Trans. Image Process..

[18] Nascimento J.M.P., Dias J.M.B. (2004). Vertex Component Analysis: A Fast Algorithm to Unmix Hyperspectral Data. IEEE Trans. Geosci. Remote Sens..

[19] Winter M.E. (1999). N-FINDR: An Algorithm for Fast Autonomous Spectral End-Member Determination in Hyperspectral Data. Int. Soc. Opt. Photonics.

[20] Heinz D.C. (2001). Fully constrained least squares linear spectral mixture analysis method for material quantification in hyperspectral imagery. IEEE Trans. Geosci. Remote Sens..

[21] Deán-Ben X.L., Deliolanis N.C., Ntziachristos V., Razansky D. (2014). Fast unmixing of multispectral optoacoustic data with vertex component analysis. Opt. Lasers Eng..

[22] Dolet A., Varray F., Mure S., Grenier T., Liu Y., Yuan Z., Tortoli P., Vray D. (2018). Spatial and Spectral Regularization to Discriminate Tissues Using Multispectral Photoacoustic Imaging. EURASIP J. Adv. Signal Process..

[23] Pratt W.K. (1978). Digital Image Processing.

[24] Yuan M., Lin Y. (2006). Model selection and estimation in regression with grouped variables. J. R. Stat. Soc. Ser. B (Stat. Methodol.).

[25] Scharf L.L. (1991). Statistical Signal Processing: Detection, Estimation, and Time Series Analysis.

[26] Plaza A., Chein-I C. (2005). An Improved N-FINDR Algorithm in Implementation. Proceedings of the Algorithms and Technologies for Multispectral, Hyperspectral, and Ultraspectral Imagery XI.

[27] Arthuis C.J., Novell A., Raes F., Escoffre J., Lerondel S., Pape A.L., Bouakaz A., Perrotin F. (2017). Real-Time Monitoring of Placental Oxygenation during Maternal Hypoxia and Hyperoxygenation Using Photoacoustic Imaging. PLoS ONE.

[28] Dolet A., Varray F., Roméo E., Dehoux T., Vray D. (2017). Spectrophotometry and Photoacoustic Imaging: A Comparative Study. IRBM.

[29] Boggio K., Nicoletti G., Carlo E.D., Cavallo F., Landuzzi L., Melani C., Giovarelli M., Rossi I., Nanni P., Giovanni C.D. (1998). Interleukin 12-mediated prevention of spontaneous mammary adenocarcinomas in two lines of Her-2/neu transgenic mice. J. Exp. Med..

[30] Visualsonics (2018). Oxy-Hemo Mode. https://www.visualsonics.com/product/software/oxy-hemo-mode.

[31] Li C., Wang L.V. (2009). Photoacoustic Tomography and Sensing in Biomedicine. Phys. Med. Biol..

[32] Deán-Ben X.L., Bay E., Razansky D. (2014). Functional Optoacoustic Imaging of Moving Objects Using Microsecond-Delay Acquisition of Multispectral Three-Dimensional Tomographic Data. Sci. Rep..

[33] Su R., Ermilov A.S., Liopo A.V., Oraevsky A.A. Optoacoustic 3D visualization of changes in physiological properties of mouse tissues from live to postmortem. Proceedings of the Photons Plus Ultrasound: Imaging and Sensing 2012.

[34] Gehrung M., Bohndiek S.E., Brunker J. (2019). Development of a blood oxygenation phantom for photoacoustic tomography combined with online pO_2_ detection and flow spectrometry. J. Biomed. Opt..

[35] Gröhl J., Kirchner T., Adler T.J., Hacker L., Holzwarth N., Hernández-Aguilera A., Herrera M.A., Santos E., Bohndiek S.E., Maier-Hein L. (2021). Learned spectral decoloring enables photoacoustic oximetry. Sci. Rep..

